# Impact of migraine on quality of life and disability: a matched cross-sectional comparative study in a tertiary hospital in Malaysia

**DOI:** 10.3389/fneur.2026.1919937

**Published:** 2026-08-24

**Authors:** Firyal Harlina Hussain, Juen Kiem Tan, Rathika Rajah, Ching Soong Khoo, Wan Nur Nafisah Wan Yahya, Rabani Remli, Michelle Maryanne Tan, Rosnah Sutan, Hui Jan Tan

**Affiliations:** 1 Department of Medicine, Faculty of Medicine, the National University of Malaysia, Kuala Lumpur, Malaysia; 2 Department of Medicine, Hospital Canselor Tuanku Muhriz, Kuala Lumpur, Malaysia; 3 Department of Medicine, Hospital Tengku Ampuan Rahimah, Klang, Selangor, Malaysia; 4 Department of Public Health Medicine, Faculty of Medicine, the National University of Malaysia, Kuala Lumpur, Malaysia

**Keywords:** MIDAS, migraine, migraine disability, quality of life, WHOQOL-BREF

## Abstract

**Background:**

Migraine-related disabilities have a significant disease burden and are an important factor in impaired quality of life (QOL) of affected persons. This study aimed to compare QOL and migraine disability score in patients with migraine with healthy controls.

**Methods:**

A matched cross-sectional comparative study was carried out at the Neurology Clinic between August 2023 and March 2026. The study population included 100 migraine patients aged 18–60 years and 100 healthy controls matched by age and sex. All participants completed the World Health Organization Quality of Life Brief (WHOQOL-BREF) questionnaire, and migraine patients completed the Migraine Disability Assessment (MIDAS).

**Results:**

A total of 100 migraine patients and 100 controls were recruited. Participants with migraine had a significantly lower total WHOQOL-BREF score than controls (*p* = 0.013). Similarly, physical health (64.86 vs. 74.52, *p* < 0.001) and psychological health scores (70.12 vs. 73.95, *p* = 0.010) were significantly lower than controls. Poor physical health was associated with QOL (*p* < 0.001, OR = 0.938). 19 percent of patients had severe disability, with a higher monthly frequency of headaches (mean 4.21). Photophobia, higher frequency of headaches, and poor overall perception of health were associated with migraine disability. The Wald statistic (11.805) suggested that headache frequency contributed meaningfully to disability. Migraine patients with disability had significantly lower total QOL scores (69.62) compared to those with no or little disability (75.00, *p* = 0.003).

**Conclusion:**

Migraines are associated with a significant decrease in QOL and increased disability, especially in physical health. These findings highlight the burden of migraine on daily function and wellbeing. Using these validated assessment tools to evaluate quality of life and detect disabilities, clinicians can facilitate more effective and individualized management plans.

## 1 Introduction

Migraine is a primary headache disorder characterized by recurrent, unilateral, debilitating headache accompanied by nausea, vomiting, photophobia and phonophobia. It can be classified by the International Classification of Headache Disorders into migraine with aura and migraine without aura (1). The global burden of migraine has escalated, with a prevalence rising from 721.9 million in 1990 to 1.16 billion in 2021 (2). In the Global Burden of Disease Study in 2019, migraine remains second among the world's causes of disability in men and women and all ages (3). It is common among adults aged 20–50 years and is 3–4 times more frequent in women than in men. The global prevalence in women is 20.7% and 9.7% in men (3). A community- based study in Malaysia reported a prevalence rate of 9% (4).

Migraine remains underestimated, underdiagnosed, and undertreated despite its common occurrence. The World Health Organization defined quality of life as an individual's perception of their position in life in the context of the culture and value systems in which they live and in relation to their goals, expectations, standards and concerns (5). People with migraine usually report poor QOL during attack-free periods more frequently than healthy controls in a local study in Malaysia (6). Migraineurs report poorer subjective wellbeing and reduced health-related quality of life (HRQOL) (7). Migraines significantly reduce the QOL, predominantly in young patients, those with frequent migraine attacks, those not using preventive medications and those suffering from chronic diseases (8).

Migraine patients suffer from significant disability with personal, economic and social burdens (9). Migraine ranks second among the world's causes of disability and the top cause of disability-adjusted life years (DALYs) in young women (10). Migraine was associated with short- and long-term employment disability and overall migraine related disability (11). A systematic review concluded that there was an increased pattern of migraine-related disability with rising headache frequency (12).

To capture the burden of migraine, validated instruments such as WHOQOL-BREF and the Migraine Disability Assessment (MIDAS) are essential. The World Health Organization Quality of Life questionnaire is one of the most frequently used generic instruments for assessing chronic disorders (13). The WHO Quality of Life Brief Questionnaires **(**WHOQOL-BREF) measures broad functional domains, while MIDAS measures headache-related disability and improve doctor-patient communication regarding the functional consequences of the disease. The Migraine Disability Assessment (MIDAS) instrument was developed to assess headache-related disability (14).

The association between migraine, quality of life and functional impairment has been well-documented in the Western population. However, a critical knowledge gap exists on how these dynamics manifest within the unique demographic and environmental landscapes of Southeast Asia. Malaysia represents a highly diverse, multi-ethnic country with distinct dietary habits, and its tropical climate may exacerbate migraine attacks.

Furthermore, existing regional literature has predominantly examined quality of life and functional disability as isolated entities. There is a lack of data employing simultaneous, multi- variable modeling to explore how distinct domains of generic health-related QOL interface with migraine-specific disability scores, particularly when captured through culturally and linguistically validated local instruments (WHOQOL-BREF and MIDAS). It remains unclear which specific clinical or sensory features drive severe functional impairment within this healthcare framework, or how incremental changes in attack frequency quantitatively accelerate the transition into severe disability.

To bridge these gaps, this study provides a localized analysis of the independent predictors of QOL and disability. By identifying these specific clinical profiles and health-system implications, this manuscript provides necessary real-world evidence to guide targeted, individualized therapeutic protocols and resource allocation within Southeast Asian tertiary care settings.

## 2 Materials and methods

### 2.1 Study design and study population

This matched cross-sectional comparative study was conducted between August 24^th^, 2023, and March 23^rd^, 2026, at the Hospital Canselor Tuanku Muhriz, National University of Malaysia. This study was conducted following the guidelines of the Declaration of Helsinki, with informed written consent obtained from each participant. Study approval was obtained from the National University of Malaysia Ethics and Research Board of the Faculty of Medicine (research code: JEP 2023-577) and funding was provided by the National University of Malaysia (FF-2023-276). The study population was recruited via simple random sampling of patients between 18 and 60 years old, with a diagnosis of migraine for at least 12 months before the study. The diagnosis of migraine was made once the diagnostic criteria stipulated in the International Headache Society (IHS) International Classification of Headache Disorders for migraine were fulfilled (1). Healthy controls were recruited on a 1:1 ratio using a hospital-based convenience sampling method, drawing from hospital staff, patient visitors and relatives accompanying patients to non-neurology clinics. Controls were matched to cases by sex and age (+/– 5 years). A healthy control was clinically defined as an individual with no history of primary or secondary headache disorders.

Patients and controls were excluded from the study if they had secondary headache disorders such as structural brain abnormalities (including traumatic brain injury, brain tumor, stroke, venous sinus thrombosis), meningoencephalitis, neurodegenerative disorders, preexisting psychiatric illness (depression or anxiety disorders), comorbid chronic pain disorders (fibromyalgia, backpain), sleep disorders (insomnia) or pregnancy.

### 2.2 Recruitment and data collection

The patients were selected based on specific inclusion criteria. The data collected included sociodemographic characteristics (age, sex, employment status, occupation, and marital status), lifestyle factors (smoking and alcohol consumption), and comorbidities. Clinical characteristics included migraine duration, associated symptoms, precipitating factors, use of abortive and prophylactic medications, monthly attack frequency, and pain severity.

Migraine was classified based on the International Classification of Headache Disorders 3^rd^ edition guidelines for headache disorders. The visual analog scale (VAS) was used to assess the pain perception of the participants during the peak of their migraine attacks. The pain score was measured on a scale of 0–10, with 0 = no pain at all and 10 = most extreme pain. Participants were required to complete WHOQOL-BREF questionnaire to measure health-related quality of life. Additionally, participants with migraine were required to complete MIDAS-M questionnaires for assessment of migraine disability. All questionnaires have been validated in the Malay language, and permission from the respective authors has been obtained.

### 2.3 WHO quality of life brief questionnaires (WHOQOL-BREF)

The WHOQOL-BREF questionnaire is a short and simple instrument for measuring health-related QOL over 2 weeks (13). This self-administered questionnaire is widely accepted for generic use in a variety of cultural settings (15). It is a 26-item abbreviated version of the World Health Organization QOL questionnaire and is regarded as very important for assessing QOL. The questionnaire consists of two parts. Part 1 includes two questions, which included an individual's overall perception of QOL and overall perception of health. Part 2 comprises 24 questions that contribute to four domain scores, reflecting an individual's perception of QOL. The WHOQOL-BREF questionnaire has four domains (physical health, psychological health, social relationships, and environment), whose scores collectively depict the QOL profile of participants. The WHOQOL-BREF uses a 5-point Likert response format with six sets of response categories (e.g., 1 “Not at all” to 5 “completely” or 1 “Very dissatisfied” to 5 “Very satisfied”). According to the original WHOQOL-BREF article, raw scores for each domain are manually calculated and then converted to transformed scores on a 0–100 scale. The total QOL score is obtained by averaging the four domain scores. Both domain scores and the total score are scaled positively, meaning higher scores indicate a higher quality of life.

### 2.4 Migraine disability assessment (MIDAS-M)

Migraine disability is commonly measured using the Migraine Disability Assessment (MIDAS) questionnaire (14). This short, self-administered questionnaire has advantages over other instruments as it consists of five questions that focus on measuring the number of days lost at work, home, and recreational activities. This instrument is scored as follows: 0–5 indicates no or little disability, 6–10 indicates mild disability, 11–20 indicates moderate disability, and higher than 20 denotes severe disability. The original validation study showed that MIDAS had a sensitivity of around 70% and specificity of about 60%−65% for identifying patients with severe migraine-related disability when compared with physician judgment. It performed best at distinguishing severe disability but was less accurate for mild or moderate categories (16).

### 2.5 Confounding control and cohort comparability analysis

To protect the validity of clinical inferences regarding the relationship between migraine, disability and quality of life (QOL), potential confounding was systematically addressed via a framework spanning study design, strict eligibility protocols, and multivariable statistical modeling.

Source population and recruitment pathway: the source population for cases comprised adult patients at the Neurology outpatient clinic, Hospital Canselor Tuanku Muhriz. The control arm was derived from the same institution setting, utilizing a hospital- based convenience sampling approach to recruit healthy hospital staff, patient visitors, and non—neurology patient relatives.

Matching and selection procedures: structural confounding by core demographic variables was controlled at the design stage. Controls were selected utilizing a strict 1:1 individual matching mechanism based on biological sex and age (± 5 years) to cases.

Comparability Assessment and Screening Protocol: both cohorts underwent an investigator-led screening checklist prior to enrollment. Potential participants in both groups were evaluated and excluded if they presented with active confounders: pre-existing diagnosed psychiatric illnesses (depressive and anxiety disorders), comorbid chronic pain conditions (fibromyalgia, low back pain), sleep disorders or neurodegenerative diseases.

Handling of residual confounding: despite matching and screening, residual confounding may stem from unmatched baseline demographic or clinical variations. Baseline balance was checked using univariate comparative testing (Table 1), which confirmed that the cohorts did not differ significantly in employment status, smoking or alcohol use. However, there were statistical differences observed in occupational profiles, marital status, hypertension and dyslipidemia. Multivariable logistic regression models were constructed to determine the true independent association of migraine parameters. These models entered potential confounders including medical comorbidities, clinical characteristics and medication profiles, thereby statistically neutralizing their capacity to bias final QOL and disability estimates.

**Table 1:** Demographic characteristics.

Characteristics	Non-migraine *N* = 100 (%)	Migraine *N* = 100 (%)	*p*-value (1-tailed)
Gender			0.500^a^
Male	9 (9.0)	9 (9.0)	
Female	91 (91.0)	91 (91.0)	
Race			0.442^a^
Malay	86 (86.0)	82 (82.0)	
Chinese	8 (8.0)	10 (10.0)	
Indian	5 (5.0)	7 (7.0)	
Punjabi	1 (1.0)	1 (1.0)	
Employment			0.214^a^
No	17 (17.0)	13 (13.0)	
Yes	83 (83.0)	87 (87.0)	
Occupation			**0.030** ^**a**^
Unemployed	17 (17.0)	13 (13.0)	
Healthcare sector	63 (63.0)	51 (51.0)	
Education sector	4 (4.0)	12 (12.0)	
Others	16 (16.0)	24 (24.0)	
Marital status			**0.036** ^**a**^
Single	26 (26.0)	27 (27.0)	
Married	74 (74.0)	68 (68.0)	
Divorced	0 (0.0)	5 (5.0)	
Smoking			0.689^b^
No	98 (98.0)	98 (98.0)	
Yes	2 (2.0)	2 (2.0)	
Alcohol			0.249^b^
No	98 (98.0)	100 (100.0)	
Yes	2 (2.0)	0 (0.0)	
Comorbidities			
Hypertension			**0.038** ^**a**^
No	95 (95.0)	88 (88.0)	
Yes	5 (5.0)	12 (12.0)	
Diabetes			**0.030** ^**b**^
No	95 (95.0)	100 (100.0)	
Yes	5 (5.0)	0 (0.0)	
Dyslipidemia			**0.006** ^**a**^
No	93 (93.0)	81 (81.0)	
Yes	7 (7.0)	19 (19.0)	
Asthma			0.222^b^
No	98 (98.0)	95 (95.0)	
Yes	2 (2.0)	5 (5.0)	
Overall perception of QOL			**0.020** ^**a**^
1-Very poor	0 (0.0)	1 (100.0)	
2-Poor	0 (0.0)	3 (100.0)	
3-Neutral	14 (14.0)	26 (26.0)	
4-Good	61 (61.0)	54 (54.0)	
5-Very good	25 (25.0)	16 (16.0)	
Overall perception of health			**< 0.001** ^**a**^
1-Very dissatisfied	0 (0.0)	1 (1.0)	
2-Dissatisfied	0 (0.0)	15 (15.0)	
3-Neutral	28 (28.0)	35 (35.0)	
4-Satisfied	62 (62.0)	43 (43.0)	
5-Very satisfied	10 (10.0)	6 (6.0)	
Mean, SD (CI, lower-upper)			
Age	39.70 ± 9.25 (37.86–41.53)	40.30 ± 9.42 (38.43–42.17)	0.325^c^
Domain 1 (Physical health)	74.52 ± 10.87 (72.36–76.68)	64.86 ± 14.34 (62.01–67.71)	**< 0.001** ^**c**^
Domain 2 (Psychological)	73.95 ± 9.39 (72.09–75.81)	70.12 ± 13.36 (67.47–72.77)	**0.010** ^**c**^
Domain 3 (Social relationship)	75.27 ± 12.32 (72.82–77.72)	73.04 ± 15.52 (69.96–76.12)	0.131^c^
Median (25^th^,75^th^ percentiles)			
Domain 4 (Environment)	75.00 (75.00, 81.00)	75.00 (69.00, 88.00)	0.372^d^
Total QOL score	74.25 (69.00, 79.75)	72.00 (64.00, 79.75)	**0.013** ^**d**^

*p*-value significant at < 0.05 (bold), SD, standard deviation; CI, confidence interval; Percentiles, (25th,75th); a, Pearson Chi-Square; *b*, Fisher's Exact Test; *c*, Independent *T*-test; d, Mann-Whitney *U*-test.

### 2.6 Statistical analysis

The sample size for this study was calculated based on the sample size by Bernard Rosner. Fundamentals of Biostatistics (5^th^ edition) (17). Based on study by Alders et al. (4).


$$\begin{array}{ccc} n & = & \frac{z^{2}xp\left(1-p\right)}{d^{2}} \end{array}$$

*z*^2^ = 1.96^2^

*p* = 0.09

*d* = 0.05


$$\begin{array}{l} \text{Sample size }=\frac{1.96^{2}\;x\;0.09\;\left(1-0.09\right)}{0.05^{2}}\\ \;=\frac{0.3146}{0.0025}\\ \;=125 \end{array}$$

The sample size estimated for this study is 125 per arm.

Data entry and analysis were done using the statistical program IBM SPSS Version 30 [International Business Machines Corporations (IBM), Singapore]. We present categorical variables such as gender, race, employment, occupation, marital status, smoking, alcohol, comorbidities, clinical features, and overall perception of QOL and health as frequencies and percentages. Testing for normality was performed using skewness and kurtosis on all continuous variables (age, frequency of pain per month, severity of pain, all 4 domains of QOL questionnaire, years of migraine and total QOL score). Normally distributed data was presented as mean, standard deviation (confidence interval, lower-upper), while non-normally distributed data were presented as median, and percentiles (25^th^, 75^th^) for the skewed data. For continuous variables, the Pearson's chi-square test, Fisher's exact test, Independent *T*-test and Mann-Whitney U test were used whenever applicable. Multiple logistic regression was used to analyze predictors of QOL between migraine and non-migraine and the risk of migraine disability across variables. Statistical significance was set at *p* < 0.05. No missing data were encountered during the analysis.

## 3 Results

Of 125 screened participants, 100 with migraine (cases) and 100 without migraine (controls) were recruited. The rest were excluded as they did not fulfill the inclusion criteria. The participants were between 20 and 60 years of age, with a mean age of 39.70 ± 9.25 years (95% CI: 37.86–41.53) for the control group and 40.30 ± 9.42 years (95% CI: 38.43–42.17) for the cases. Most participants were female (91; 91%) for both cases and controls. Mostly were Malay with (86, 86%), (82; 82%) for non-migraine and migraine patients, respectively. Most participants in both groups were employed, with 83.0% of the non-migraine group and 87.0% of the migraine group. Gender, race, employment status, smoking and alcohol did not differ significantly between the groups (*p* > 0.05). The sociodemographic data of this cohort are presented in Table 1.

A significant difference was observed in occupational categories (*p* = 0.030), where a higher proportion of migraine participants worked in the education sector (12.0%) and other occupations (24.0%) compared with the non-migraine group (63.0% and 16.0%, respectively). Most participants were married (74.0% in the non-migraine group and 68.0% in the migraine group), followed by single individuals (26.0% and 27.0%, respectively). The comorbidities that were more common in the migraine group compared to controls were hypertension (12% vs. 5%, *p* = 0.038) and dyslipidemia (19% vs. 7%, *p* = 0.006).

Overall perception of QOL differed significantly between groups (*p* = 0.020). A greater proportion of participants in the non-migraine group reported good or very good QOL compared with those in the migraine group. Similarly, overall perception of health status showed a statistically significant difference between groups (*p* < 0.001), with a higher proportion of migraine participants reporting dissatisfaction with their health compared to the non-migraine group.

In the physical health domain (Domain 1), participants without migraine demonstrated significantly higher mean QOL scores, 74.52 ± 10.87 (95% CI: 72.36–76.68), compared to those with migraine, 64.86 ± 14.34 (95% CI: 62.01–67.71), (*p* < 0.001). Similarly, in the psychological domain (Domain 2), the non-migraine group reported significantly higher mean scores, 73.95 ± 9.39; 95% CI: 72.09–75.81, compared with the migraine group, 70.12 ± 13.36; 95% CI: 67.47–72.77, (*p* = 0.010). indicating poorer psychological wellbeing among migraine participants. The social relationship domain (Domain 3) showed the non-migraine group with a mean score of 75.27 ± 12.32 (95% CI: 72.82–77.72), compared with 73.04 ± 15.52 (95% CI: 69.96–76.12) in the migraine group. In the environmental domain (Domain 4), the median score for the non-migraine group was 75.00 (25^th^–75^th^ percentile: 75.00–81.00), while the migraine group had a median score of 75.00 (25^th^–75^th^ percentile: 69.00–88.00). There was a statistically significant difference between the groups in the social relationship and environment domain. The overall total QOL score was significantly higher in the non-migraine group, with a median of 74.25 (25^th^–75^th^ percentile: 69.00–79.75), compared with 72.00 (25^th^–75^th^ percentile: 64.00–79.75) in the migraine group (*p* = 0.013). Overall, these findings indicate that migraine is associated with significantly lower quality of life, particularly in the physical health and psychological domains.

Table 2 demonstrates the clinical features of migraine with various disabilities. Increased migraine disability is associated with higher headache frequency, 4.21 ± 1.54 (95% CI: 3.46–4.96), greater pain severity, 6.84 ± 1.77 (95% CI: 5.99–7.70), poorer physical, 55.37 ± 14.26 (95% CI: 48.50–62.24) and psychological QOL scores, 65.84 ± 14.20 (95% CI: 59.00–72.69) and less favorable perceptions of health. Sociodemographic differences such as occupation (42.1%), as well as comorbid dyslipidemia (26.3%), also appear more common in patients with severe disability. These findings underscore the cumulative burden of migraine on both clinical outcomes and quality of life.

**Table 2:** Clinical features of migraine patients with various disability (*N* = 100).

Characteristics	No/little disability *N* = 44 (%)	Mild disability *N* = 23 (%)	Moderate disability *N* = 14 (%)	Severe disability *N* = 19 (%)
Gender				
Male	4 (9.1)	0 (0.0)	0 (0.0)	5 (26.3)
Female	40 (90.9)	23 (100.0)	14 (100.0)	14 (73.7)
Race				
Malay	33 (75.0)	21 (91.3)	10 (71.4)	18 (94.7)
Chinese	5 (11.4)	1 (4.3)	3 (21.4)	1 (5.3)
Indian	5 (11.4)	1 (4.3)	1 (7.1)	0 (0.0)
Punjabi	1 (2.3)	0 (0.0)	0 (0.0)	0 (0.0)
Employment				
No	6 (13.6)	4 (17.4)	2 (14.3)	1 (5.3)
Yes	38 (86.4)	19 (82.6)	12 (85.7)	18 (94.7)
Occupation				
Unemployed	6 (13.6)	4 (17.4)	2 (14.3)	1 (5.3)
Healthcare sector	27 (61.4)	9 (39.1)	8 (57.1)	7 (36.8)
Education sector	4 (9.1)	4 (17.4)	1 (7.1)	3 (15.8)
Others	7 (15.9)	6 (26.1)	3 (21.4)	8 (42.1)
Marital				
Single	10 (22.7)	7 (30.4)	3 (21.4)	7 (36.8)
Married	32 (72.7)	15 (65.2)	10 (71.4)	11 (57.9)
Divorced	2 (4.5)	1 (4.3)	1 (7.1)	1 (5.3)
Smoking				
No	44 (100.0)	23 (100.0)	14 (100.0)	17 (89.5)
Yes	0 (0.0)	0 (0.0)	0 (0.0)	2 (10.5)
Comorbidities				
Hypertension				
No	36 (81.8)	22 (95.7)	11 (78.6)	19 (100.0)
Yes	8 (18.2)	1 (4.3)	3 (21.4)	0 (0.0)
Dyslipidemia				
No	36 (81.8)	20 (87.0)	11 (78.6)	14 (73.7)
Yes	8 (18.2)	3 (13.0)	3 (21.4)	5 (26.3)
Asthma				
No	41 (93.2)	22 (95.7)	13 (92.9)	19 (100.0)
Yes	3 (6.8)	1 (4.3)	1 (7.1)	0 (0.0)
Presence of aura				
No	15 (34.1)	8 (34.8)	4 (28.6)	7 (36.8)
Yes	29 (65.9)	15 (65.2)	10 (71.4)	12 (63.2)
Photophobia				
No	6 (13.6)	6 (26.1)	3 (21.4)	8 (42.1)
Yes	38 (86.4)	17 (73.9)	11 (78.6)	11 (57.9)
Phonophobia				
No	21 (47.7)	12 (52.2)	6 (42.9)	8 (42.1)
Yes	23 (52.3)	11 (47.8)	8 (57.1)	11 (57.9)
Character of pain				
Pulsating	18 (40.9)	12 (52.2)	5 (35.7)	9 (47.4)
Throbbing	25 (56.8)	11 (47.8)	8 (57.1)	10 (52.6)
Both	1 (2.3)	0 (0.0)	1 (7.1)	0 (0.0)
Anatomical site				
Frontal	16 (36.4)	7 (30.4)	4 (28.6)	8 (42.1)
Occipital	3 (6.8)	1 (4.3)	1 (7.1)	1 (5.3)
Parietal	11 (25.0)	6 (26.1)	2 (14.3)	2 (10.5)
Temporal	14 (31.8)	9 (39.1)	7 (50.0)	8 (42.1)
Side of pain				
Unilateral	32 (72.7)	20 (87.0)	13 (92.9)	13 (68.4)
Bilateral	12 (27.3)	3 (13.0)	1 (7.1)	6 (31.6)
Precipitating factors				
Noise/sound				
No	21 (47.7)	12 (52.2)	6 (42.9)	8 (42.1)
Yes	23 (52.3)	11 (47.8)	8 (57.1)	11 (57.9)
Light				
No	14 (31.8)	12 (52.2)	4 (28.6)	10 (52.6)
Yes	30 (68.2)	11 (47.8)	10 (71.4)	9 (47.4)
Sunlight				
No	15 (34.1)	10 (43.5)	4 (28.6)	8 (42.1)
Yes	29 (65.9)	13 (56.5)	10 (71.4)	11 (57.9)
Coffee				
No	32 (72.7)	16 (69.6)	10 (71.4)	13 (68.4)
Yes	12 (27.3)	7 (30.4)	4 (28.6)	6 (31.6)
Tea				
No	42 (95.5)	21 (91.3)	14 (100.0)	17 (89.5)
Yes	2 (4.5)	2 (8.7)	0 (0.0)	2 (10.5)
Food				
No	41 (93.2)	21 (91.3)	11 (78.6)	14 (73.7)
Yes	3 (6.8)	2 (8.7)	3 (21.4)	5 (26.3)
Types of food				
Chocolate	2 (4.5)	1 (4.3)	2 (14.3)	1 (5.3)
Peanut	0 (0.0)	0 (0.0)	0 (0.0)	1 (5.3)
Salty food	1 (2.3)	1 (4.3)	0 (0.0)	1 (5.3)
MSG containing food	0 (0.0)	0 (0.0)	0 (0.0)	2 (10.5)
Carbonated drink	0 (0.0)	0 (0.0)	1 (7.1)	0 (0.0)
Not affected by food	41 (93.2)	21 (91.3)	11 (78.6)	14 (73.7)
Abortive medication				
PCM				
No	16 (36.4)	13 (56.5)	7 (50.0)	5 (26.3)
Yes	28 (63.6)	10 (43.5)	7 (50.0)	14 (73.7)
NSAIDs				
No	26 (59.1)	11 (47.8)	6 (42.9)	9 (47.4)
Yes	18 (40.9)	12 (52.2)	8 (57.1)	10 (52.6)
Sumatriptan				
No	35 (79.5)	17 (73.9)	9 (64.3)	17 (89.5)
Yes	9 (20.5)	6 (26.1)	5 (35.7)	2 (10.5)
Cafergot				
No	40 (90.9)	21 (91.3)	12 (85.7)	16 (84.2)
Yes	4 (9.1)	2 (8.7)	2 (14.3)	3 (15.8)
Antiemetics				
No	42 (95.5)	23 (100.0)	13 (92.9)	19 (100.0)
Yes	2 (4.5)	0 (0.0)	1 (7.1)	0 (0.0)
Prophylactic medications				
B- blockers				
No	35 (79.5)	18 (78.3)	8 (57.1)	12 (63.2)
Yes	9 (20.5)	5 (21.7)	6 (42.9)	7 (36.8)
Calcium antagonist				
No	39 (88.6)	19 (82.6)	13 (92.9)	17 (89.5)
Yes	5 (11.4)	4 (17.4)	1 (7.1)	2 (10.5)
Antidepressant				
No	38 (86.4)	20 (87.0)	12 (85.7)	14 (73.7)
Yes	6 (13.6)	3 (13.0)	2 (14.3)	5 (26.3)
Antiseizure				
No	41 (93.2)	23 (100.0)	10 (71.4)	17 (89.5)
Yes	3 (6.8)	0 (0.0)	4 (28.6)	2 (10.5)
CGRP Antagonist				
No	43 (97.7)	23 (100.0)	14 (100.0)	18 (94.7)
Yes	1 (2.3)	0 (0.0)	0 (0.0)	1 (5.3)
Overall perception of QOL				
1-Very poor	1 (2.3)	0 (0.0)	0 (0.0)	0 (0.0)
2-Poor	0 (0.0)	1 (4.3)	0 (0.0)	2 (10.5)
3-Neither poor nor good	9 (20.5)	7 (30.4)	4 (28.6)	6 (31.6)
4-Good	26 (59.1)	12 (52.2)	6 (42.9)	10 (52.6)
5-Very good	8 (18.2)	3 (13.0)	4 (28.6)	1 (5.3)
Overall perception of health				
1-Very dissatisfied	1 (2.3)	0 (0.0)	0 (0.0)	0 (0.0)
2-Dissatisfied	2 (4.5)	5 (21.7)	3 (21.4)	5 (26.3)
3-Neither dissatisfied nor satisfied	10 (22.7)	10 (43.5)	3 (21.4)	12 (63.2)
4-Satisfied	29 (65.9)	7 (30.4)	5 (35.7)	2 (10.5)
5-Very satisfied	2 (4.5)	1 (4.3)	3 (21.4)	0 (0.0)
Mean, SD (CI, lower-upper)				
Age	41.50 ± 9.90 (38.48–44.51)	41.08 ± 9.41 (37.01–45.15)	39.64 ± 11.36 (33.08–46.20)	37.05 ± 6.06 (34.12–39.97)
Frequency of pain per month	2.41 ± 1.43 (1.97–2.85)	3.22 ± 0.73 (2.90–3.54)	3.93 ± 1.32 (3.16–4.70)	4.21 ± 1.54 (3.46–4.96)
Severity of pain	6.02 ± 1.94 (5.43–6.61)	6.43 ± 1.72 (5.69–7.18)	6.64 ± 1.82 (5.59–7.70)	6.84 ± 1.77 (5.99–7.70)
Domain 1 (physical health)	70.64 ± 14.09 (66.35–74.92)	61.61 ± 10.83 (56.92–66.29)	64.93 ± 12.89 (57.48–72.37)	55.37 ±14.26 (48.50–62.24)
Domain 2 (psychological)	73.36 ± 13.50 (69.26–77.47)	67.17 ± 10.78 (62.51–71.84)	70.57 ± 14.26 (62.33–78.81)	65.84 ± 14.20 (59.00–72.69)
Domain 3 (social relationship)	76.98 ± 13.87 (72.76–81.20)	68.48 ± 13.82 (62.50–74.46)	71.79 ± 11.13 (65.36–78.21)	70.37 ±21.67 (59.92–80.81)
Median (25^th^,75^th^ Percentiles)				
Years of migraine	9.50 (5.00, 17.00)	9.00 (3.00, 15.00)	4.50 (3.00, 17.50)	7.00 (5.00, 18.00)
Domain 4 (Environment)	81.00 (75.00, 88.00)	75.00 (63.00, 88.00)	75.00 (65.75, 88.00)	75.00 (63.00, 88.00)
Total QOL score	75.00 (69.00, 81.43)	67.50 (62.75, 73.50)	71.25 (65.25, 79.56)	69.00 (53.25, 79.50)

SD, standard deviation; CI, confidence interval; Percentiles, (25th, 75th).

Table 3 presents the clinical characteristics of migraine patients by disability level (no or little disability vs. mild to severe disability). Rational for categorization: MIDAS cut-offs (0–5, 6–10, 11–20, >20) are standard; collapsing into binary (0–5 vs. ≥6) is widely used to distinguish clinically meaningful disability. Among the 100 participants, 44 (44.0%) had no or little disability, while 56 (56.0%) had mild to severe disability. Most participants in both groups were female (90.9% vs. 91.1%). Gender, race, employment status, occupation, marital status, smoking status and comorbidities (hypertension, dyslipidaemia, asthma) were not significantly associated with migraine disability (*p* > 0.05).

**Table 3:** Clinical features of migraine patients with no/little disability and mild to severe disability.

Characteristics	No/little disability *N* = 44 (%)	Mild to severe disability *N* = 56 (%)	*p*-value (1-tailed)
Gender			0.622^b^
Male	4 (9.1)	5 (8.9)	
Female	40 (90.9)	51 (91.1)	
Race			0.129^a^
Malay	33 (75.0)	49 (87.5)	
Chinese	5 (11.4)	5 (8.9)	
Indian	5 (11.4)	2 (3.6)	
Punjabi	1 (2.3)	0 (0.0)	
Employment			0.433^a^
No	6 (13.6)	7 (12.5)	
Yes	38 (86.4)	49 (87.5)	
Occupation			0.112^a^
Unemployed	6 (13.6)	7 (12.5)	
Healthcare sector	27 (61.4)	24 (42.9)	
Education sector	4 (9.1)	8 (14.3)	
Others	7 (15.9)	17 (30.4)	
Marital status			0.331^a^
Single	10 (22.7)	17 (30.4)	
Married	32 (72.7)	36 (64.3)	
Divorced	2 (4.5)	3 (5.4)	
Smoking			0.311^b^
No	44 (100.0)	54 (96.4)	
Yes	0 (0.0)	2 (3.6)	
Comorbidities			
Hypertension			0.085^b^
No	36 (81.8)	52 (92.9)	
Yes	8 (18.2)	4 (7.1)	
Dyslipidemia			0.426^a^
No	36 (81.8)	45 (80.4)	
Yes	8 (18.2)	11 (19.6)	
Asthma			0.386^b^
No	41 (93.2)	54 (96.4)	
Yes	3 (6.8)	2 (3.6)	
Presence of aura			0.493^a^
No	15 (34.1)	19 (33.9)	
Yes	29 (65.9)	37 (66.1)	
Photophobia			**0.024** ^**a**^
No	6 (13.6)	17 (30.4)	
Yes	38 (86.4)	39 (69.6)	
Phonophobia			0.448^a^
No	21 (47.7)	26 (46.4)	
Yes	23 (52.3)	30 (53.6)	
Character of pain			0.427^a^
Pulsating	18 (40.9)	26 (46.4)	
Throbbing	25 (56.8)	29 (51.8)	
Both	1 (2.3)	1 (1.8)	
Anatomical site			0.339^a^
Frontal	16 (36.4)	19 (33.9)	
Occipital	3 (6.8)	3 (5.4)	
Parietal	11 (25.0)	10 (17.9)	
Temporal	14 (31.8)	24 (42.9)	
Side of pain			0.129^a^
Unilateral	32 (72.7)	46 (82.1)	
Bilateral	12 (27.3)	10 (17.9)	
Precipitating factors			
Noise/sound			0.448^a^
No	21 (47.7)	26 (46.4)	
Yes	23 (52.3)	30 (53.6)	
Light			0.069^a^
No	14 (31.8)	26 (46.4)	
Yes	30 (68.2)	30 (53.6)	
Sunlight			0.296^a^
No	15 (34.1)	22 (39.3)	
Yes	29 (65.9)	34 (60.7)	
Coffee			0.368^a^
No	32 (72.7)	39 (69.6)	
Yes	12 (27.3)	17 (30.4)	
Tea			0.460^b^
No	42 (95.5)	52 (92.9)	
Yes	2 (4.5)	4 (7.1)	
Food			0.090^b^
No	41 (93.2)	46 (82.1)	
Yes	3 (6.8)	10 (17.9)	
Types of food			0.281^a^
Chocolate	2 (4.5)	4 (7.1)	
Peanut	0 (0.0)	1 (1.8)	
Salty food	1 (2.3)	2 (3.6)	
MSG containing food	0 (0.0)	2 (3.6)	
Carbonated drink	0 (0.0)	1 (1.8)	
Unaffected by food	41 (93.2)	46 (82.1)	
Abortive medication			
Paracetamol			0.201^a^
No	16 (36.4)	25 (44.6)	
Yes	28 (63.6)	31 (55.4)	
NSAIDs			0.104^a^
No	26 (59.1)	26 (46.4)	
Yes	18 (40.9)	30 (53.6)	
Sumatriptan			0.370^a^
No	35 (79.5)	43 (76.8)	
Yes	9 (20.5)	13 (23.2)	
Cafergot			0.418^b^
No	40 (90.9)	49 (87.5)	
Yes	4 (9.1)	7 (12.5)	
Antiemetics			0.410^b^
No	42 (95.5)	55 (98.2)	
Yes	2 (4.5)	1 (1.8)	
Prophylactic medications			
Beta- blockers			0.095^a^
No	35 (79.5)	38 (67.9)	
Yes	9 (20.5)	18 (32.1)	
Ca antagonist			0.431^a^
No	39 (88.6)	49 (87.5)	
Yes	5 (11.4)	7 (12.5)	
Antidepressant			0.284^a^
No	38 (86.4)	46 (82.1)	
Yes	6 (13.6)	10 (17.9)	
Antiseizures			0.378^b^
No	41 (93.2)	50 (89.3)	
Yes	3 (6.8)	6 (10.7)	
CGRP antagonist			0.689^b^
No	43 (97.7)	55 (98.2)	
Yes	1 (2.3)	1 (1.8)	
Overall perception of QOL			0.135^a^
1-Very poor	1 (2.3)	0 (0.0)	
2-Poor	0 (0.0)	3 (5.4)	
3-Neutral	9 (20.5)	17 (30.4)	
4-Good	26 (59.1)	28 (50.0)	
5-Very good	8 (18.2)	8 (14.3)	
Overall perception of health			**< 0.001** ^**a**^
1-Very dissatisfied	1 (2.3)	0 (0.0)	
2-Dissatisfied	2 (4.5)	13 (23.2)	
3-Neutral	10 (22.7)	25 (44.6)	
4-Satisfied	29 (65.9)	14 (25.0)	
5-Very satisfied	2 (4.5)	4 (7.1)	
Mean, SD (CI, lower-upper)			
Age	41.5 ± 9.90 (38.48–44.51)	39.3 ± 9.00 (36.94–41.76)	0.131^c^
Frequency of pain per month	2.41 ± 1.43 (1.97–2.85)	3.73 ± 1.27 (3.39–4.07)	**< 0.001** ^**c**^
Severity of pain	6.02 ± 1.94 (5.43–6.61)	6.63 ± 1.74 (6.16–7.09)	0.053^c^
Domain 1 (Physical health)	70.64 ± 14.09 (66.35–74.92)	60.32 ± 12.93 (56.86–63.79)	**< 0.001** ^**c**^
Domain 2 (Psychological)	73.36 ± 13.50 (69.26–77.47)	67.57 ± 12.80 (64.14–71.00)	**0.015** ^**c**^
Domain 3 (Social relationship)	76.98 ± 13.87 (72.76–81.20)	69.95 ± 16.16 (65.62–74.27)	**0.012** ^**c**^
Median (25^th^,75^th^ Percentiles)			
Years of migraine	9.50 (5.00,17.00)	7.00 (3.00,16.50)	0.189^d^
Domain 4 (Environment)	81.00 (75.00,88.00)	75.00 (63.00,88.00)	**0.032** ^**d**^
Total QOL score	75.00 (69.00,81.43)	69.62 (58.31,78.18)	**0.003** ^**d**^

*p*-value significant at < 0.05 (bold), SD, standard deviation; CI, confidence interval; Percentiles, (25^th^,75^th^), a, Pearson Chi-Square; b, Fisher's Exact Test; c, Independent *T*-test; d, Mann-Whitney *U* test.

Among the migraine symptoms, photophobia showed a statistically significant association with migraine disability (*p* = 0.024). However, the presence of aura, phonophobia, character of pain, anatomical site of pain, and side of pain were not significantly associated with migraine disability. Precipitating factors such as noise/sound, light, sunlight exposure, coffee, tea, and certain foods (chocolate, peanuts, salty food, monosodium glutamate containing food, carbonated drinks), did not show statistically significant differences between the two disability groups (*p* > 0.05). The use of abortive medications (paracetamol, non-steroidal anti-inflammatory drugs (NSAIDs), sumatriptan, cafergot, and antiemetics) showed no statistically significant association with migraine disability. Likewise, the use of prophylactic medications, including beta-blockers, calcium channel antagonists, antidepressants, antiseizures, and CGRP antagonists, did not differ significantly between the two groups. Regarding overall QOL perception, no significant difference was observed between the two groups. However, overall perception of health showed a statistically significant association with migraine disability (*p* < 0.001), where patients with mild to severe disability reported lower levels of satisfaction with their health.

Analysis of continuous variables showed that frequency of pain per month was significantly higher among patients with mild to severe disability, 3.73 ± 1.27 (95% CI: 3.39–4.07), compared to those with no/little disability, 2.41 ± 1.43 (95% CI: 1.97–2.85) (*p* < 0.001). In contrast, there was no significant difference in age and pain severity between the groups (*p* = 0.131 and *p* = 0.053, respectively).

With respect to QOL domains, patients with mild to severe disability had significantly lower scores in Domain 1 (Physical health) 60.32 ± 12.93 (95% CI: 56.86–63.79) (*p* = < 0.001), Domain 2 (Psychological) 67.57 ± 12.80 (95% CI: 64.14–71.00) (*p* = 0.015), Domain 3 (Social relationships) 69.95 ± 16.16 (95% CI: 65.62–74.27) (*p* = 0.012), in compare to no/little disability with 70.64 ± 14.09 (95% CI: 66.35–74.92), 73.36 ± 13.50 (95% CI: 69.26–77.47) and 76.98 ± 13.87 (95% CI: 72.76–81.20), respectively.

For non-normally distributed data, Domain 4 (Environment) scores were significantly lower among patients with mild to severe disability, with a median score of 75.00 (25^th^; 63.00, 75^th^; 88.00) compared with those with no/little disability 81.00 (25^th^; 75.00, 75^th^; 88.00), (*p* = 0.032). The total QOL score was significantly lower in the mild to severe disability group 69.62 (25^th^; 58.31, 75^th^; 78.18) compared to the no/little disability group 75.00 (25^th^; 69.00, 75^th^; 81.43), (*p* = 0.003). However, years of migraine did not significantly differ between the two groups (*p* = 0.189). Overall, the findings indicate that photophobia, frequency of pain per month, perceived health status, and lower QOL scores across several domains were significantly associated with greater migraine disability.

Table 4 demonstrates the regression model highlights that photophobia (*B* = −1.400, SE = 0.690, Wald = 4.124, df = 1, Odds ratio = 0.247, *p* = 0.042) and poor perceived health (*B* = −0.046, SE = 0.022, Wald = 4.449, df = 1, Odds ratio = 0.955, *p* = 0.035) were significant independent correlates of migraine disability, while sociodemographic factors and comorbidities did not show significant associations. Frequency of pain per month was entered as a categorical variable, but the regression output showed unstable coefficients and extremely large odds ratios with wide confidence intervals, reflecting model instability. Despite this, the Wald statistic (11.805) suggested that headache frequency contributed meaningfully to disability, consistent with descriptive findings in Table 2. These findings reinforce the role of sensory symptoms and subjective health perception in shaping functional impairment among migraine patients.

**Table 4:** Multiple logistic regression analysis for association of migraine disability in migraine patients.

Variables	*B*	S.E.	Wald	df	*P*-value	Exp (B)	95% C.I.	
							Lower	Upper
Marital Status	−0.060	0.593	0.010	1.000	0.920	0.942	0.295	3.012
Occupation	0.550	0.291	3.579	1.000	0.059	1.733	0.980	3.064
Hypertension	−0.968	0.945	1.048	1.000	0.306	0.380	0.060	2.423
Dyslipidemia	0.592	0.762	0.603	1.000	0.437	1.807	0.406	8.045
Photophobia	−1.400	0.690	4.124	1.000	**0.042**	0.247	0.064	0.952
Frequency of pain per month			11.805	7.000	0.107			
< 1/month	0.367	32,110.192	0.000	1.000	1.000	1.443	0.000	
1/month	19.908	15,214.554	0.000	1.000	0.999	4.42 x 10^8^	0.000	
2/month	22.049	15,214.554	0.000	1.000	0.999	37.63 x 10^8^	0.000	
>2/month	22.210	15,214.554	0.000	1.000	0.999	44.21 x 10^8^	0.000	
>5/month	22.181	15,214.554	0.000	1.000	0.999	42.97 x 10^8^	0.000	
>10/month	21.153	15,214.554	0.000	1.000	0.999	15.36 x 10^8^	0.000	
>15/month	42.022	27,143.969	0.000	1.000	0.999	17.7 x 10^17^	0.000	
Overall perception of health (Q2)	−0.046	0.022	4.449	1.000	**0.035**	0.955	0.915	0.997

B, regression coefficient; S.E., standard error; Wald, Wald chi-square statistic; df, degrees of freedom; Exp (B), odds ratio; C.I., confidence interval.

## 4 Discussion

### 4.1 Sociodemographic and regional characteristics

This research helps to address an important gap in our regional clinical data, which explores how migraine influences quality of life and contributes to disability among our local population in the following sections. This research provides critical regional evidence that highlights the unique clinical drivers of migraine burden within a Southeast Asian framework. While classical demographic associations like female preponderance align with global trends, the novelty of our data emerges from the simultaneous modeling of socioenvironmental factors and metabolic comorbidities within this specific population. The treatment of migraine does not rely solely on abortive clinical pain management. It requires a comprehensive approach addressing lifestyle stressors, metabolic health and specific environmental triggers.

This study found migraine was more prevalent in women, consistent with findings from numerous epidemiological studies, likely due to hormonal influences and psychosocial factors. The mean age of migraine participants was 40.30 ± 9.42 years, which is consistent with reports indicating that migraine most often affects individuals during the most productive phases of their lives (18). The Global Burden of Disease Study identified migraine as the leading cause of years lived with disability worldwide among individuals aged 15–49 years, affecting both men and women (19). Furthermore, in 2019, migraine remained a major contributor to the global loss of healthy life, as measured by disability -adjusted life years (DALYs), particularly among young adult women (10). The relationship between employment and social habits and migraine has remained controversial. Contrary to a previous study which found that migraine risk was significantly associated with unemployment and smoking (20), our findings did not find any significant association with these variables. However, Tang et al. (21) reported a significant negative association between dietary alcohol intake and migraine. Although there were no significant association between migraine and race, employment status, smoking, or alcohol consumption, further research is required to evaluate these variables and their potential roles in migraine pathology.

The higher proportion of migraine participants in education and other occupations may reflect occupational stress as a trigger, consistent with studies linking psychosocial stressors to migraine. Marital status differences, particularly the presence of divorce among migraine participants, suggest that chronic migraine may strain interpersonal relationships, echoing findings that migraine negatively affects family and social functioning (22).

Migraine have been linked to the traditional risk factors for cardiovascular events (18, 22, 23). The higher prevalence of hypertension and dyslipidemia among migraine patients supports evidence that migraine is associated with vascular risk factors, potentially increasing long-term cardiovascular burden. Huang et al. reported that individuals with migraine demonstrated an increased risk of atherosclerotic cardiovascular disease, compared to those without migraine (24). A study among US adult population showed that migraine was associated with a higher risk of cardiovascular disease, and these findings highlight that migraine plays an important role in cardiovascular disease (25).

These comorbidities may act synergistically with migraine pathophysiology, increasing the risk of stroke, ischemic heart disease, and vascular complications. Possible mechanisms suggest that migraine is associated with generalized endothelial dysfunction (26) and increased platelet aggregation (27). This supports the need for comprehensive management strategies that address both migraine symptoms and vascular risk factors, including lifestyle modification, cardiovascular screening, and careful selection of migraine therapies.

### 4.2 Health-system implications and tool utility

The distinct value of utilizing linguistically and culturally adapted versions of the WHOQOL -BREF and MIDAS- M lies in their capacity to reveal the hidden aspects in health systems. The finding of physical health domain acts as the independent predictor of overall QOL suggests that subjective patient wellbeing is strongly linked to physical productivity and functional preservation. These data provide a clear direction for health systems to improve management of early preventive migraine care.

### 4.3 QOL and domains between migraine and non-migraine

Overall perception of QOL and health was significantly poorer among migraine participants, consistent with global studies showing that migraine reduces subjective wellbeing and satisfaction with health. The quality of life in migraine patients in different parts of the world showed a decline in the quality of life. A study in Saudi Arabia reported that QOL in migraine was moderate, with females having poorer QOL compared to males (28). In Italy, migraine patients had lower health -related QOL scores than the general population and worsened consistently with increased migraine severity (29). Similarly, a Malaysian study also demonstrated that female migraine patients had experienced significantly lower QOL than the control group (6). Patients often develop a negative mindset about their health, reporting feelings of vulnerability, reduced confidence in coping, and persistent worry about future attacks. This altered perception contributes to a poorer quality of life even during attack-free periods (30).

In the physical health domain, the migraine group had a lower mean score compared to the non-migraine group. This finding indicates that physical functioning is the area most severely affected, reflecting how migraine attacks disrupt daily activities and reduce productivity. The pattern observed here aligns with broader evidence showing that migraine often leads to limitations in physical roles and contributes significantly to disability worldwide (10, 31). In a national US survey, 53% reported that headaches caused bed rest, 31% missed at least 1 day of work or school, and 51% reported a reduction in work or school productivity (32). These impairments extend beyond physical limitations, manifesting as a significant reduction in workplace performance and productivity, highlighting migraine's pervasive impact on overall quality of life.

The psychological domain was also significantly affected, highlighting the emotional burden of migraine (22). Lower psychological scores indicate diminished wellbeing, as individuals experience reduced resilience, coping capacity, and overall life satisfaction. This decline directly translates into a poorer quality of life, since psychological functioning is a critical determinant of how patients manage the recurrent and disabling nature of migraine. Lopez-Medina D et al. reported 38.6% showed cognitive impairment among migraine patients who completed the Mini-Mental State Examination (30). Chronic migraine is strongly linked to poor mental health, reduced cognition, and lower quality of life (30, 33). From meta-synthesis of qualitative studies, migraine affects all aspects of life, including personal, social, work, and emotional health, and many patients feel stigmatized because others often struggle to understand their condition (34). Depression, anxiety and emotional distress are frequently reported among migraine sufferers and contribute to diminished coping capacity and social withdrawal. These findings concurred with a previous study, which found that anxiety and depression were associated with migraine (35). The bidirectional relationship between migraine and psychological wellbeing suggests that interventions targeting mental health may yield improvements in overall QOL.

This study demonstrated that physical health is the most robust independent predictor of QOL among individuals with migraine. Although both physical and psychological domains are conceptually important, the results revealed that only physical health showed statistical significance. This finding suggests that the impact of psychological health may be mediated through physical functioning, or that its contribution overlaps with the physical domain. The odds ratio further highlights the strength of this association; poorer physical health substantially reduced the likelihood of better QOL outcomes. These findings align with prior research indicating that physical limitations due to migraine, such as fatigue, pain, and reduced functional capacity are central to the burden experienced by patients. Patients with migraine had worse health-related QOL than the matched general population control group (31, 36). Shaik et al. reported that female migraine patients had lower physical health compared to healthy controls (6). Taken together, these findings confirm that migraine substantially impairs quality of life, with physical health emerging as the strongest independent predictor, while psychological health contributes to the overall burden but not as a unique determinant. This reinforces the need for interventions that prioritize physical functioning such as pain management and strategies to improve daily activity levels.

In this study, migraine sufferers had statistically significantly lower median total QOL scores. Consistently, total QOL, physical health and psychological health scores were significantly lower among migraine participants than nonmigraine controls from several studies. A retrospective analysis of National Health and Wellness Survey data showed that migraine patients with ≥4 monthly headache days and more treatment failures experienced greater burden, impaired quality of life, and reduced functioning (37). Shaik et al. reported that migraine patients with lower total QOL scores had 1.2 times higher odds of having disability than patients with higher total QOL scores in one of tertiary hospital in Malaysia (6). Similar findings were demonstrated in studies from United States (38), Arab Saudi (28) and Japan (39). These data across diverse populations in North America, Europe and Asia reinforce the status of migraine as a major disabling condition with a profound global burden.

These findings underscore the need for holistic management strategies that address not only pain control but also psychological support and lifestyle modification. By integrating routine screening for psychological distress, tailored behavioral interventions, and patient education, quality of life outcomes can be enhanced. Moreover, workplace accommodations and public health initiatives targeting stress reduction could mitigate occupational triggers, particularly in high-risk sectors, reinforcing the importance of a comprehensive biopsychosocial approach to migraine management.

### 4.4 Migraine disability.

The present study highlights several clinical features and quality of life (QOL) correlates associated with migraine disability. Among the migraine participants, 56.0% experienced mild to severe disability, underscoring the substantial burden of migraine in this population. While demographic variables such as gender, race, employment, occupation, marital status, and comorbidities were not significantly associated with disability, certain clinical and symptomatic features emerged as important contributors.

Photophobia emerged as a predictive factor for migraine disability. This finding concurred with results from the American Migraine Registry for Migraine Research, which reported photophobia was associated with reduced work productivity and migraine-related disability (40). The sensory hypersensitivity symptoms of photophobia frequently overlap with phonophobia and osmophobia, and they were reported to have a significant impact on headache-related disability (41). This finding points to the complex role that photophobia plays in migraine. Sensitivity to light is a hallmark symptom that often intensifies the experience of an attack (42). The clinician's role in assessing disability is complex, as the impact may not be immediately apparent. Many patients adopt coping strategies such as avoiding bright environments or using tinted lenses to mitigate discomfort. The results emphasize the importance of comprehensive and systematic investigations into the complex interplay between photophobia and migraine to improve our understanding and tailor our management for patients with migraine.

Although other clinical features, such as aura, pain character, and medication usage, did not differ significantly, the trend toward greater use of prophylactic medications among more disabled patients suggests a higher disease burden requiring more intensive management. Individuals with more disabling migraine attacks are more likely to be prescribed prophylactic medications such as β blockers and antidepressants, as these therapies aim to reduce attack frequency, severity, and the overall impact on daily functioning (43, 44). This pattern suggests that prophylactic treatment is not merely a clinical choice but a marker of higher disease burden. Patients with mild or infrequent attacks may rely on acute therapies alone, whereas those with significant impairment require long-term preventive strategies (45). Thus, the presence of prophylactic medication use in a patient population can be interpreted as an indicator of greater disability and reduced quality of life (16).

Self-rated health was a predictor of migraine disability, indicating that patients who perceived their health as poorer were more likely to experience disability. The most reported psychosocial difficulties among migraine patients included emotional problems, poorer physical and mental health, limited social functioning, and greater overall disability (46). This finding is consistent emphasizing the subjective burden of migraine, where perceived health status often mediates the relationship between clinical symptoms and functional outcomes. Migraine is an important poor self-rated health factor (47). Poorer self-rated health may reflect cumulative effects of comorbidities, psychological distress, and reduced coping capacity, all of which contribute to disability. The finding highlights the importance of incorporating patient-reported outcomes into migraine management, as they provide valuable insights into the lived experience of disability.

The frequency of migraine attacks per month is associated with disability. This aligns with previous studies demonstrating that attack frequency is the most consistent determinant of migraine-related disability and reduced QOL (22). The frequency of migraine was associated both with increased MIDAS score and with employment disability (11). A cross-sectional study from Saudi Arabia reported that patients who had less frequent migraine attacks exhibited better QOL (28). Health-related quality of life improves when patients experience fewer headache days with effective prophylaxis; therefore, initiating preventive treatment early is essential to minimize quality of life decline and reduce the overall burden of migraine (16). A study from Malaysia, showed 73% of patients experienced severe disability, with significantly higher number of days with headaches (13.8 days/3 months) and pain scores (6). Similarly, a systemic review and meta-analysis showed pattern of increasing migraine-related disability with increasing headache frequency (28). Frequent attacks disrupt daily functioning, reduce productivity, and increase healthcare utilization, highlighting the importance of preventive strategies aimed at reducing attack frequency.

Taken together, these results emphasize the multifactorial nature of migraine-related disability. Frequency of migraine attacks per month, sensory symptoms such as photophobia and subjective health perceptions also contribute meaningfully. Management strategies should not only aim to reduce attack frequency but also address sensory triggers and enhance patients' overall health perception. The WHOQOL BREF measures broad functional domains, while MIDAS focuses specifically on headache related disability. The integration of these questionnaires with pharmacological treatment, lifestyle modifications, patient education, and psychosocial support may therefore yield the greatest improvements in reducing disability and improving quality of life.

## 5 Conclusion

Migraine is associated with significantly reduced quality of life and increased disability, particularly in the domains of physical health. Poor physical health was a predictor of reduced QOL. Headache frequency, photophobia, and subjective health perceptions emerging as key predictors. Every additional headache day is associated with more than a two-fold increase in disability. These findings highlight the substantial burden of migraine on daily functioning and wellbeing. By using these validated assessment tools to evaluate quality of life and screen for disability, healthcare professionals can achieve a better understanding of migraine pathology and ultimately facilitate more effective and individualized management plans.

## 6 Limitation

The study is limited by its matched cross-sectional design, which precludes drawing causal inferences between migraine characteristics and long-term changes in quality of life. Second, the reliance on a single center cohort and a hospital-based convenience sampling method poses potential bias, as individuals within a hospital framework may display distinct healthcare seeking behaviors compared to the community. The residual confounding remains a limitation although controls were strictly matched for age and sex. Significant baseline differences between the groups in occupation, marital distribution and metabolic conditions (hypertension and dyslipidemia) could be confounders for health-related quality of life metrics. The use of self-reported questionnaires introduces potential recall bias and subject variability. There was no subgroup analysis of the migraine participants.

## Data Availability

The datasets presented in this study can be found in online repositories. The names of the repository/repositories and accession number(s) can be found below: https://doi.org/10.6084/m9.figshare.32101102.
